# Widespread Doublecortin Expression in the Cerebral Cortex of the *Octodon degus*

**DOI:** 10.3389/fnana.2021.656882

**Published:** 2021-04-29

**Authors:** Thomas van Groen, Inga Kadish, Natalija Popović, María Caballero Bleda, Beatriz Baño-Otalora, María Angeles Rol, Juan Antonio Madrid, Miroljub Popović

**Affiliations:** ^1^Department of Cell Biology, University of Alabama at Birmingham, Birmingham, AL, United States; ^2^Department of Human Anatomy and Psychobiology, Faculty of Medicine, University of Murcia, Murcia, Spain; ^3^Institute of Biomedical Research of Murcia, Virgen de la Arrixaca University Hospital, University of Murcia, Murcia, Spain; ^4^CEIR Campus Mare Nostrum (CMN), University of Murcia, Murcia, Spain; ^5^Division of Neuroscience and Experimental Psychology, Faculty of Biology Medicine and Health, University of Manchester, Manchester, United Kingdom; ^6^Chronobiology Laboratory, Department of Physiology, Faculty of Biology, University of Murcia, Murcia, Spain

**Keywords:** *Octodon degus*, doublecortin, cerebral cortex, limbic system, cortical atlas

## Abstract

It has been demonstrated that in adulthood rodents show newly born neurons in the subgranular layer (SGL) of the dentate gyrus (DG), and in the subventricular zone (SVZ). The neurons generated in the SVZ migrate through the rostral migratory stream (RMS) to the olfactory bulb. One of the markers of newly generated neurons is doublecortin (DCX). The degu similarly shows significant numbers of DCX-labeled neurons in the SGL, SVZ, and RMS. Further, most of the nuclei of these DCX-expressing neurons are also labeled by proliferating nuclear antigen (PCNA) and Ki67. Finally, whereas in rats and mice DCX-labeled neurons are predominantly present in the SGL and SVZ, with only a few DCX neurons present in piriform cortex, the degu also shows significant numbers of DCX expressing neurons in areas outside of SVZ, DG, and PC. Many areas of neocortex in degu demonstrate DCX-labeled neurons in layer II, and most of these neurons are found in the limbic cortices. The DCX-labeled cells do not stain with NeuN, indicating they are immature neurons.

## Introduction

The diurnal rodent *Octodon degus* has been found to be suitable model to investigate diurnal rhythm (Jiao et al., 1999; Ocampo-Garcés et al., 2005; Hummer et al., 2007; Vivanco et al., 2007, 2010a, b; Otalora et al., 2010; Bonmati-Carrion et al., 2017; Baño-Otálora et al., 2020), cognitive functions (Okanoya et al., 2008; Popović et al., 2010; Uekita and Okanoya, 2011; Pereda-Pérez et al., 2013; Tarragon et al., 2014), social interactions (Braun et al., 2003; Ziabreva et al., 2003; Lee, 2004; Poeggel et al., 2005; Colonnello et al., 2011), age-related neuropathology, and behavioral alterations (Inestrosa et al., 2005; Popović et al., 2009; van Groen et al., 2011; Braidy et al., 2012) as well as hippocampal neurogenesis (Kumazawa-Manita et al., 2013a; Akers et al., 2014). Akers et al. (2014) demonstrated modest reduction in proliferating Ki67^+^ cells and immature doublecortin (DCX) positive neurons in dentate gyrus (DG) of infant degus and guinea pigs in comparison to mice. This modest reduction in hippocampal neurogenesis was related to slower infant forgetting in precocial rodents (degus and guinea pigs). Kumazawa-Manita et al. (2013a), using bromodeoxyuridine (BrdU) (a marker of newly generated cells) and polysialylated-neural cell adhesion molecule (PSA-NCAM) (a marker of immature neurons) demonstrated that tool use training but not radial maze increases neurogenesis in the DG of adult degus.

With the exception of two cetacean species (Northern minke whale and harbor porpoise), in most mammals examined thus far, the subgranular layer (SGL) of the DG and the subventricular zone (SVZ) give rise to new neurons in the adult (review see Taupin and Gage, 2002; Patzke et al., 2015; Lipp, 2017), including humans (Eriksson et al., 1998; Bhardwaj et al., 2006; Knoth et al., 2010). Variations in extent of adult neurogenesis, and natural and experimental factors controlling it have been described in laboratory animals (Amrein et al., 2004). A commonly used marker for newly born neurons, DCX, labels the newly born neurons in the SGL and SVZ. Furthermore, it has been demonstrated that in some species, i.e., rabbit (Luzzati et al., 2009), tenrec (Alpár et al., 2010), giant otter shrew (Patzke et al., 2013), guinea pig (Xiong et al., 2008), Megachiropteran and Microchiropteran bats (Chawana et al., 2013, 2016, 2020), four-striped mice (Olaleye and Ihunwo, 2014) three prosimian primates: Demidoff’s dwarf bushbaby, the potto, and the ring-tailed lemur (Fasemore et al., 2018), and four afrotherian mammals: hottentot golden mole, the rock hyrax, the eastern rock sengi, and the four-toed sengi (Patzke et al., 2014), neuronal DCX expression is not confined to DG and SVZ. In these species, DCX-labeled neurons have been described in many cortical and subcortical areas, e.g., amygdala, piriform, and limbic cortices. In general, the DCX-positive neurons are found in limbic cortical areas, but their expression is not limited to limbic regions of the brain. The wide range of variation seen between species, raises the question as to where and how much these DCX labeled neurons are present in other rodents (Amrein et al., 2004; Schauwecker, 2006) and if they are newly born neurons (Nacher et al., 2001). To address this issue, we investigated the brain of *O. degus* using markers for proliferating cells, i.e., proliferating nuclear antigen (PCNA), Ki67 (von Bohlen und Halbach, 2007, 2011), and a marker for developing neurons, DCX.

## Materials and Methods

### Animals

Female *O. degus* were used at the age of 1 year (*n* = 6). The lifespan of the degu in captivity can be up to 14 years (Lee, 2004), but most animals only reach 5–7 years of age. In contrast, the lifespan of the degu in the wild is only quite short, less than 50% reach 1 year of age, and only 1% reach 2 years of age (Fulk, 1976). Reproductive female period starts between 6 and 9 months and drops off after 4 to 4.5 years of age (Lee, 2004), together indicating the female degu used in the present study could be considered as young adult animals. The animals were obtained from a colony maintained at the Animal Service of the University of Alicante. Animals were housed in Plexiglas cages in an isolated room (Chronolab), with controlled humidity (60%), temperature (23 ± 1°C) and under a 12:12 light/dark cycle (light on from 07:00 to 19:00 h). Light was provided by fluorescent lamps controlled by an electronic timer (Data Micro, Orbis), with a light intensity of 350–400 lux at the level of the cages. The degus were fed *ad libitum* throughout the experiment, using commercial rat chow (A04, rat-mouse maintenance, Panlab). All procedures were conducted in accordance with the local Institutional Animal Care and Use Committee (IACUC) guidelines and followed the guidelines of the European Communities Council Directive of 24 November 1986 (86/609/EEC). The protocol was approved by the Local Committee on the Ethics of Animal Experiments. All efforts were made to minimize animal suffering.

### Fixation and Tissue Preparation

In short, the degus were deeply anesthetized with sodium pentobarbital (Ovation Pharmaceuticals, Deerfield, IL, United States, 70 mg/kg, i.p.) and perfused transcardially with phosphate buffered saline (PBS) followed by 4% paraformaldehyde in 0.1 M phosphate buffer (pH 7.4). The brains were removed from the skull and postfixed for 4 h in the same fixative, at 4°C. Then, the brains were transferred to a 30% sucrose solution in PBS overnight at 4°C and day after were stored in antifreeze (15% sucrose with 30% ethylene glycol).

Following washing in 30% sucrose, the brains were cut using a freezing, sliding microtome in 30 μm thick coronal sections (six series: 1 in 6), which were collected in PBS (100 mM, pH 7.4).

### Immunohistochemistry

One half of the first series of sections was mounted unstained for Nissl staining, the other half was stained for (1/4) DCX (goat anti-DCX; Santa Cruz, CA, United States, sc-8066; Naritsuka et al., 2009) and 1/4 for PCNA (goat anti-PCNA; Santa Cruz, CA, United States, sc-9857; Martínez-Navarrete et al., 2008) Similarly, 1/4 of the second series was stained for Ki67 (rabbit anti-Ki67; Novocastra, Buffalo Grove, IL, United States, NCL-Ki67p; Baker et al., 2006), according to published protocols (Kadish et al., 2002). The last 1/4 of the second series was used for double-staining experiments. The other series were stored at −20°C in antifreeze for future analysis. The sections destined for PCNA staining were pretreated for 30 min with hot (85°C) citrate buffer. In short, the series of sections were transferred to a solution containing the primary antibody, this solution consists of TBS with 0.5% Triton X-100 added (TBS-T). Following incubation in this solution for 18 h (overnight) on a shaker table at room temperature (20°C), the sections were rinsed three times in TBS-T and transferred to the solution containing the secondary antibody (rabbit anti-goat Ig × biotin; Thermo Scientific or goat anti-rabbit Ig × biotin; Millipore). After 2 h, the sections were rinsed three times with TBS-T and transferred to a solution containing mouse ExtrAvidin^®^ (Sigma), following rinsing the sections were incubated for approximately 3 min with Ni-enhanced DAB (Kadish et al., 2002). All stained sections were mounted on slides and coverslipped with DPX.

In a selected set of 50 sections, the cells were double-labeled for DCX and Ca^2+^ binding proteins [i.e., calretinin, calbindin, and parvalbumin (rabbit; Swant, Marly, Switzerland; Huesa et al., 2008)] following a similar protocol with appropriately labeled fluorescent secondary antibodies. Similarly sections were double-labeled for DCX and NeuN (Neuronal Nuclei, Chemicon, Temecula, CA, United States, MAB377; Mullen et al., 1992; Vazdarjanova et al., 2006), similarly, with appropriately labeled fluorescent secondaries. Finally, all stained sections were mounted on slides and coverslipped.

### Specificity of the Primary Antibodies

All antibodies that were used in this study are commercially available, and have been shown to be specific for the appropriate antigen in both the mouse and the rat brain in our hands (Supplementary Data 1).

### Image Analysis

The staining patterns of DCX, PCNA, and Ki67 were analyzed using low-power images, the cytoarchitectonic borders of cortical areas were established according to the adjacent Nissl-stained section. The nomenclature was taken from the previously described neuroanatomical regions of the degu (Wright and Kern, 1992; Kumazawa-Manita et al., 2013b). Digital photomicrographs were captured using DP70 camera (Olympus) using Cellsense software (Olympus). To avoid changes in lighting, which might affect measurements, all images were acquired in one session. Further, to avoid differences in staining density between sections, the measurements were performed on sections that were stained simultaneously, i.e., in the same staining tray (*N* = 24). No pixilation adjustments, or manipulation of the captured images was undertaken, except for the adjustment of contrast, brightness, and levels using Cellsense software.

### Semi-Quantitative Data Analysis

For semi-quantitative evaluation of Ki67, PCNA, and DCX labeled neurons five grades were used: −, no positive neurons present; +, one or only a few cells; ++, several neurons; +++, a substantial number of neurons, ++++, the majority of the expected number of cells is positive (Verwer et al., 2007). The cortical borders were evaluated using the cytoarchitecture in cresyl violet stained sections.

## Results

### Distribution of Doublecortin Expressing Cells

**FIGURE 1:**
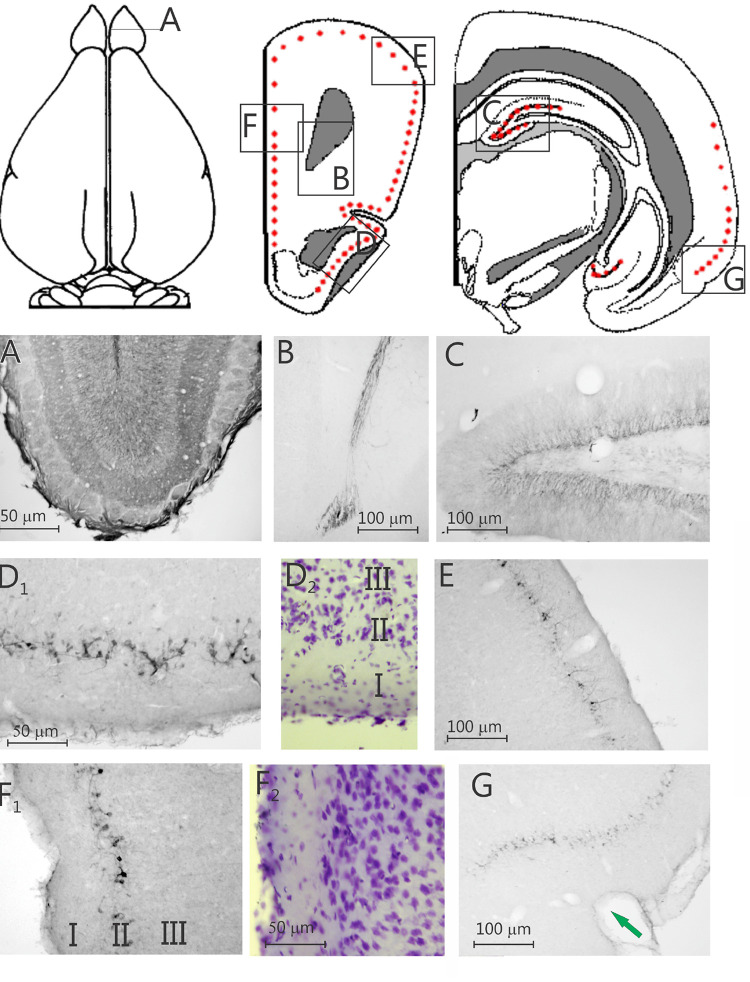
Two schematic drawings of rostral and one of caudal coronal sections, respectively, through the degu forebrain, the red dots indicate the position of doublecortin labeled cells, the boxes indicate the position of the nine photomicrographs below. Nine photomicrographs of coronal sections of a degu brain stained with doublecortin. **(A)** Olfactory bulb; **(B)** rostral migratory stream; **(C)**, dentate gyrus; **(D_1_)** piriform cortex (PC); **(D_2_)** adjacent Nissl-stained section; **(E)** dorsolateral frontal cortex; **(F_1_)** prelimbic cortex; **(F_2_)** adjacent Nissl-stained section; **(G)** perirhinal and ectorhinal cortex. Arrow in **G** indicates the border between entorhinal cortex and perirhinal cortex.

**FIGURE 2:**
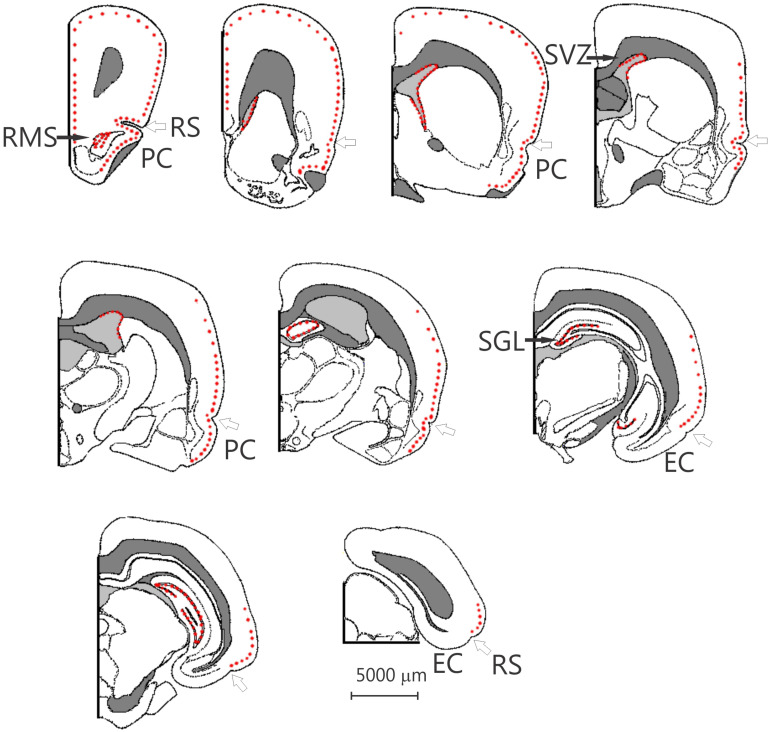
Nine schematic drawings of coronal sections of the degu brain, from rostral to caudal, the dots indicate the position of doublecortin labeled cells. The open arrow indicates the rhinal sulcus. The distance between the drawings is approximately 1.5 mm, the location of bregma is indicated by the vertical arrow. EC, entorhinal cortex; PC, piriform cortex; RMS, rostral migratory stream; RS, rhinal sulcus; SGL, subgranular layer; SVZ, subventricular zone. Modified from Wright and Kern (1992).

**FIGURE 3:**
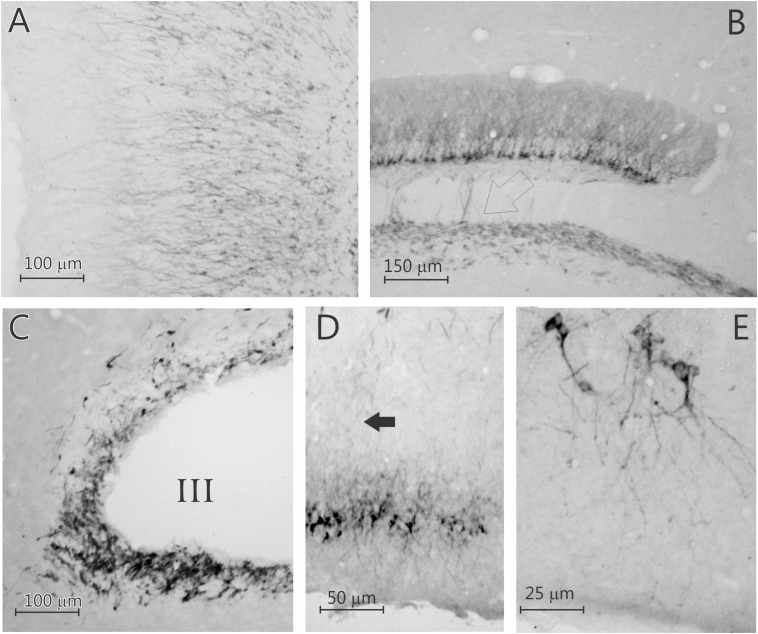
Five photomicrographs of coronal sections of the degu brain, stained for doublecortin. **(A)** Olfactory bulb; **(B)** dentate gyrus; **(C)** subventricular zone (SVZ); **(D)** caudal piriform cortex; **(E)** caudal piriform cortex. Open arrow in **(B)** indicates labeled axons in the mossy fiber bundle, arrow in **(D)** indicates labeled axons innervating the posterior piriform cortex that originated from the rostral piriform cortex. Note that the doublecortin labeled neurons in the neocortical areas are pyramidal cells.

High numbers of labeled neurons, and dense staining are present in the SGL of the DG of the hippocampus (Figures 1–3 and Supplementary Table 1). In general, the dendrites are lightly stained, and the neuronal soma are densely stained, however, some axonal staining is also present (i.e., DCX labeling in the mossy fibers, Figure 3B). Similar to the DG SGL, significant labeling is present in the SVZ; Figure 3C and Supplementary Table 1) and in the rostral migratory stream (RMS; Figure 1B and Supplementary Table 1). Further, many DCX-labeled neurons are present in the olfactory bulb (Figure 1A), and labeling for DCX is also present in the olfactory axons innervating the olfactory bulb (not illustrated).

Surprisingly, a significant number of DCX expressing neurons is evident outside the classical two areas of adult neurogenesis (i.e., SGL and SVZ), i.e., many areas of the neocortex show DCX-labeled neurons (Figures 1–3). These neurons are predominantly found in layer II of the cortex (Figures 1–3), and are more prevalent in the superficial part of layer II (Figure 1). The majority of staining is present in neurons of the limbic cortex (e.g., the prelimbic, infralimbic, and piriform cortices; Figures 1, 2), but it should be noted that, as an exception, no DCX-labeled neurons are present in the entorhinal cortex (Figure 1G). A significant number of DCX-labeled neurons is present in the olfactory cortex (i.e., rostral and caudal piriform cortex), and this is the only cortex where a few labeled neurons are also present within layer III (Figure 3E). The labeled neurons give rise to a large number of DCX-labeled axons (Figure 3D, arrow). The trajectory of the labeled axons of the piriform cortex neurons can be followed to the caudal ipsilateral piriform cortex where they likely terminate. Furthermore, the labeled axons can also be followed through the anterior commissure and these axons seem to terminate in the contralateral caudal piriform cortex (not illustrated). Finally, the DCX-labeled neurons are not limited to the limbic cortex, quite a few labeled neurons are present in the caudal temporal cortex, including the visual cortex (Figure 2). It should be noted that a very small number of labeled neurons are present in the amygdala (not illustrated), but no labeled neurons are present in any other subcortical area.

**FIGURE 4:**
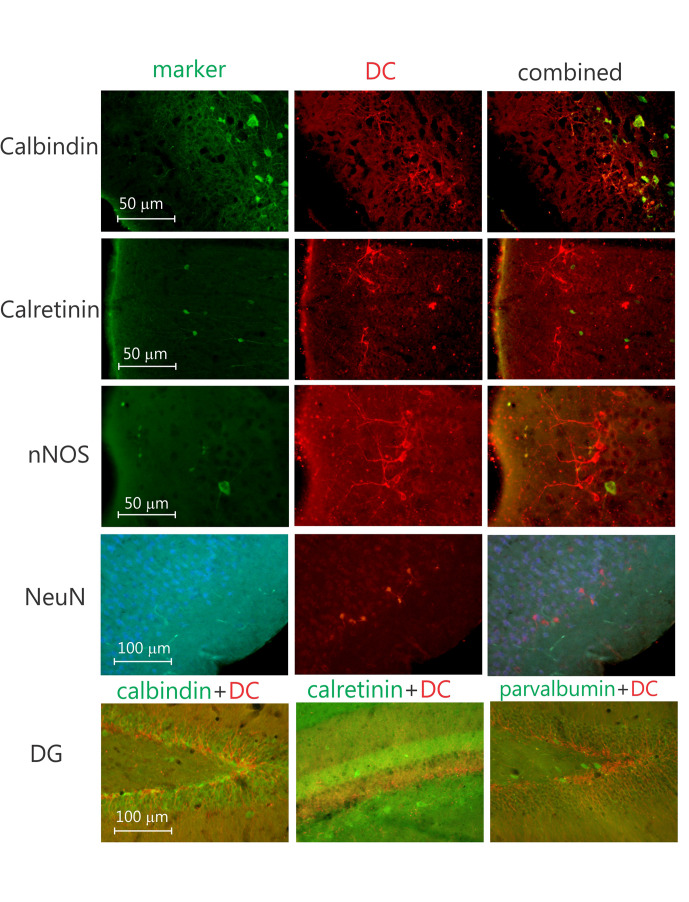
Four sets of three photomicrographs of coronal sections doublestained for neuronal markers and DCX in the piriform cortex. From top to bottom, sections stained for calbindin, calretinin, nNOS (neuronal nitric oxide), and NeuN (neuronal nucleus), respectively, please note the lack of double-labeled neurons. Bottom row, three photomicrographs of the dentate gyrus, doublestained for DCX and, parvalbumin, calretinin, and calbindin, respectively, again note the lack of doublestained neurons, indicating the DCX neurons likely are not interneurons.

In contrast to the olfactory bulb and SGL of the DG, where all labeled neurons are granular neurons (Figure 3), nearly all of the labeled neurons in the neocortex seem to be pyramidal cells (Figure 3 and Supplementary Figure 2). To investigate if any of these DCX-labeled neurons were interneurons, doublestaining with DCX and either parvalbumin, calretinin, or calbindin28D was performed. None of these revealed any double-labeled neurons (Figure 4), neither in the olfactory bulb (not illustrated), neocortex, nor DG (Figure 4). The combined staining of DCX with either GAD67 or nNOS also did not show any double-labeled neurons anywhere (not illustrated). Finally, no observable staining of DCX is present in either astrocytes, oligodendrocytes or microglial cells, neither are any DCX-labeled cells present in blood vessel walls. It should be noted that none of the DCX-labeled neurons in the hippocampus or neocortex was also labeled with either calretinin or NeuN.

The DCX-positive cells exhibited pyramidal neuron-like phenotype in layer II of the cortex (Figure 3 and Supplementary Figure 2), with relatively heterogeneous morphological characteristics (Figure 3D). Most are smaller cells, but a few larger cells are present (Figure 3). The dendrites are present in different number, length and branching complexity, but most neurons have apical dendrites extending into layer I, while the basal dendrites are present in layers II and III. Axon-like processes elongate and extend to deeper cortical layers (Figure 3D). The axons of the DCX-labeled DG granule cells extend into the mossy fiber pathway (Figure 3B).

### Distribution of PCNA and Ki67 Expressing Cells

In general, the distribution of PCNA and Ki67 labeled cells is similar to the distribution of DCX labeled neurons, especially in the DG and RMS (Supplementary Figure 1). Relatively high numbers of PCNA labeled cells are present in the SGL of the DG of the hippocampus, similarly, a large number of labeled cells is present in the RMS (Supplementary Table 1). Overall, the distribution of labeled cells in the neocortex is similar between DCX, PCNA, and Ki67.

## Discussion

Overall, our findings indicate that, similar to rats and mice, degu brains present molecular markers strongly reminiscent of neurogenesis in the SVZ and in the SGL of the DG. In contrast to rats and mice, however, degu brains also show a significant amount of DCX-labeled immature neurons in the neocortex. This suggests that the degu is another wild-type animal model to show DCX expression in widespread areas of the neocortex, similarly to the guinea pig (Xiong et al., 2010) and rabbit (Luzzati et al., 2009). However, these cells are most likely immature neurons (no Neu-N expression is present in these cells).

In the hippocampus, some of the cells that are labeled by DCX are also labeled by PCNA and Ki67, two cell cycle markers. The expression of PCNA and Ki67 has been proposed to assay cell proliferation *in situ* (Kurki et al., 1986; Valero et al., 2005). PCNA, a cofactor of DNA polymerase, is expressed during the S-phase of cell cycle (Kurki et al., 1986) while Ki67 is expressed in all phases of the cell cycle except the resting phase and a short period at the beginning of the phase G1 (Zacchetti et al., 2003). Ki67 has a very short half-life, is not detectable during DNA repair processes and is strongly downregulated, if not absent, in quiescent cells (Zacchetti et al., 2003). It has been suggested that Ki67 offers a more reliable marker to identify cells that reenter the cell cycle than PCNA (Kee et al., 2001). Consistent with these observations, we noticed significantly more labeled cell nuclei using PCNA as a marker of cell division than with Ki67.

In this study, the protocol that we followed did not allow for injection of BrdU, therefore we have used Ki67 as putative marker for proliferation. The quantification of Ki67-positive cells has been shown to reflect cellular proliferation in a manner consistent with BrdU labeling in the adult DG (Eadie et al., 2005), supporting the use of markers of cell cycle for studying adult neurogenesis. When using Ki67 to quantify cell proliferation, significantly more cells will be immunolabeled with Ki67 than by BrdU, as the former is expressed during most phases of the cell cycle and the latter labels only S-phase cells, thereby possibly allowing a better estimation of the proliferative activity (Muskhelishvili et al., 2003; Eadie et al., 2005).

As mentioned earlier, adult hippocampal neurogenesis has been reported in the vast majority of mammals investigated so far (for review, see Taupin and Gage, 2002; Patzke et al., 2015), including non-human primates (Rakic, 2002; Cai et al., 2009; Zhang et al., 2009; Jabès et al., 2010) and humans (Eriksson et al., 1998; Bhardwaj et al., 2006; Knoth et al., 2010). Only two cetacean species (Northern minke whale and harbor porpoise) appearing to lack adult neurogenesis (Patzke et al., 2015). The rate of adult neurogenesis as well as the number of proliferation sites decreases in adult mammals (Walter et al., 2009). In all mammals investigated (except possibly some bats, Amrein et al., 2007), the SGL of the DG and the SVZ give rise to new neurons, with additional reports of neurogenesis in the substantia nigra (Zhao et al., 2003) and some cortical areas in the tenrec (Alpár et al., 2010), guinea pig (Xiong et al., 2010), rabbit (Luzzati et al., 2009), cat and primate (Cai et al., 2009). We demonstrate, for the first time, adult neurogenesis in the young, adult degu SVZ, and SGL. Further, we show significant numbers of immature neurons labeled by DCX in widespread areas of the neocortex.

It has been demonstrated that within the SGL of the DG endogenous precursor cells continuously proliferate, migrate into the granule cell layer (GCL) and give rise to mature neurons which – under physiological conditions – functionally integrate into the existing hippocampal circuitry (Cameron et al., 1993; Kee et al., 2001; van Praag et al., 2002). Similarly, in the degu, the precursor cells proliferate in the SGL of the DG, migrate to their appropriate position, and most likely, integrate into the local circuitry, as indicated by the DCX-labeled mossy fibers that synapse on CA3 neurons. It is likely that the immature neocortical neurons also integrate into and/or participate in the local circuitry, as indicated by the DCX-positive axons in the caudal piriform cortex, that arise from the DCX-labeled neurons in the rostral piriform cortex. These axons from the rostral piriform cortex terminate in the appropriate area of the posterior piriform cortex (Haberly and Price, 1978).

Axons showing intracellular presence of DCX have been demonstrated before in several species. For instance, axons in olfactory nerve of the degu demonstrate the presence of DCX, as has been demonstrated previously in the rat (Nacher et al., 2001). Similarly, mossy fiber axons of the degu are labeled by DCX, as has been demonstrated in the rat and mouse (Cameron and McKay, 2001; von Bohlen und Halbach, 2007).

It has been shown that the early postmitotic stage of granule cell development during adult hippocampal neurogenesis is characterized by transient expression of calretinin (Brandt et al., 2003). Calretinin expression could be detected as early as 1 day after dividing cells were labeled with BrdU. Further, early after BrdU labeling, calretinin was colocalized with the immature neuronal marker DCX; and, at later stages of neuronal maturation, calretinin was shown to be present together with the persisting neuronal marker NeuN in the mouse (von Bohlen und Halbach, 2007). Surprisingly, in our material we did not see any double-labeling with DCX and calretinin anywhere, including the DG SGL. Most likely this is caused by the difference in species, i.e., mouse versus degu. Along those lines we also did not see any double-labeling for DCX and NeuN, indicating that the DCX-labeled neurons in the DG are not yet fully mature neurons, as has been demonstrated previously (von Bohlen und Halbach, 2007). Similarly, it has been demonstrated that during their maturation, neurons in the SGL, after starting to express DCX, express calretinin followed by calbindin (Brandt et al., 2003; Kempermann et al., 2004). Again, our material does not show any DCX neurons expressing either calretinin or calbindin. This can be caused by the difference in the expression of Ca-binding proteins between species, i.e., mouse/rat versus degu, or by a different species-specific neuronal maturation process. Most likely the neuronal maturation process is slightly different, since Ca^2+^ binding protein expression is quite similar between degu and mice (van Groen, 2001; Braun et al., 2011).

The neocortical layer II DCX cells exhibited remarkably heterogeneous yet apparently correlated morphological characteristics. They show the same anatomical characteristics as the non-DCX-labeled neurons surrounding the labeled neurons. From small to larger cells, somal shape ranges from round/oval to bipolar, including multipolar or irregular. The dendrites increase in number, length, and branching complexity; and axon-like processes elongate and extend to deeper cortical layers and contralateral cortex. These morphological variables observed across DCX cells are largely comparable to those characterized in developing cortical neurons *in vivo* and *in vitro* (del Rio et al., 1992; de Lima and Voigt, 1997). Thus, it is quite plausible that layer II DCX cells may be immature and developing neurons (Bonfanti and Nacher, 2012). Consistent with this hypothesis, the relative levels of DCX in individual cells correlate with somal size and the complexity of neuritic processes. Thus, DCX expression appears to increase as the cells become larger and more mature-looking, most likely until some peak point which is followed by downregulation of DCX expression.

Adult neurogenesis in mammals can be downregulated by age and stress (Tanapat et al., 1998; Gould and Tanapat, 1999). It has been suggested that the social environment contributes to neurogenesis, i.e., isolation decreases proliferation and “male exposure” increase cell numbers (Fowler et al., 2002). Degus are very social animals with, for instance, communal rearing of young, and are also communicating much with sound (Ziabreva et al., 2003; Lee, 2004; Poeggel et al., 2005). Recently, it has been shown that paternal recognition of their young is related to newly integrated neurons in both the olfactory bulb and the DG (Mak and Weiss, 2010). The social structure of degu colonies would likely need similar mechanisms, while this would likely explain putative new neurons in the olfactory cortices, and possibly even the limbic cortices, it is, at the present, unclear which factors cause the long-lasting neuronal immaturity in layer II of the neocortex of the degu.

## Conclusion

In conclusion, similar to other species, in degu many DCX-labeled neurons are present in DG, SVZ, RMS, and olfactory bulb. However, in contrast to laboratory mouse and rat, but similar to tenrec and guinea pig, the young, adult degu also shows significant numbers of immature DCX-labeled neurons in many areas of neocortex. In future, BrdU labeling experiments will be performed to answer questions raised in the present study.

## Data Availability Statement

The original contributions presented in the study are included in the article/Supplementary Material, further inquiries can be directed to the corresponding author/s.

## Ethics Statement

The animal study was reviewed and approved by the Institutional Animal Care and Use Committee (IACUC).

## Author Contributions

All authors participated in work planning. TvG, IK, NP, MCB, BB-O, and MP performed the experiments. TvG, IK, and MP organized the data presentations. TvG, IK, NP, and MP contributed to drafting the work. All authors have approved the final manuscript.

## Conflict of Interest

The authors declare that the research was conducted in the absence of any commercial or financial relationships that could be construed as a potential conflict of interest.
