# Supplementary material for: Widespread Doublecortin Expression in the Cerebral Cortex of the Octodon degus
Source: Front Neuroanat. 2021 Apr 29;15:656882. doi: 10.3389/fnana.2021.656882 (PMC8116662; doi:10.3389/fnana.2021.656882)
Supplement: Supplementary file 1 [file Table_1.DOCX]

**Supplementary Data S1**

**Antibodies**

All antibodies that were used in this study are commercially available, and have been shown to be specific for the appropriate antigen in both the mouse and the rat brain in our hands. The manufacturer (Swant) states that the Ca^2+-^binding antibodies bind the appropriate antigen in human, monkey, rat, mouse, guinea pig, chicken and fish, thus, most likely also in degu. Furthermore, the staining of the Ca^2+-^binding antibodies in the degu brain is very similar to previously described distributions of these proteins in the rat and mouse brain (van Groen, 2001), and in degu (Braun et al, 2010). Therefore, we have not tested the cross-reactivity of these antibodies by using antigenspecific blocking techniques in the degu.

**Specificity of the primary antibodies**

The specificity of the anti-DCX antibody (goat anti-DCX; Santa Cruz, sc-8066; Figure 1) has been characterized by: 1) preabsorption of the antibody with an excess of its immunizing peptide and 2) competitive binding assay, i.e., incubation of sections together with an excess of the immunizing peptide, both treatments eliminate all immunolabeling. Furthermore, the Santa Cruz goat anti-DCX antibody has the identical staining pattern as the rabbit anti-DCX antibody from Cell Signaling that has a different epitope on the DCX protein. Similarly, the specificity of the PCNA (rabbit anti-PCNA) and Ki67 (rabbit anti-Ki67) antibodies have been characterized by: 1) preabsorption of the antibody with an excess of its immunizing peptide, and 2) competitive binding assay, i.e., incubation of sections together with an excess of the immunizing peptide, both treatments eliminate all immunolabeling.

For negative controls, sections containing the olfactory bulb, dentate gyrus and limbic cortex have been processed for immunoreactivity with either: 1) omission of the primary antibody, 2) omission of the secondary antibody; these control stainings were negative, i.e., a complete absence of immunostaining.

In a small number of sections, the DCX labeled neurons were double-labeled for DCX and PCNA or Ki67 following a similar protocol with appropriately labeled fluorescent secondary antibodies antibodies (FITC*goat anti-rabbit & Cy3*donkey anti-goat; Jackson or Cy3*goat anti-rabbit & DyLight488 donkey anti-goat; Jackson).
