# Supplementary material for: Widespread Doublecortin Expression in the Cerebral Cortex of the Octodon degus
Source: Front Neuroanat. 2021 Apr 29;15:656882. doi: 10.3389/fnana.2021.656882 (PMC8116662; doi:10.3389/fnana.2021.656882)
Supplement: Supplementary file 2 [file Table_2.DOCX]

Supplementary Table 1

Table demonstrating the average (of 3 sections) DCX, PCNA and Ki67 labeled neurons/nuclei.

|  | **OB** | **PIR** | **PLC** | **ILC** | **IC** | **MC** | **AU** | **PR** | **EC** | **TA** | **RMS** | **SVZ** | **SGL** |
| --- | --- | --- | --- | --- | --- | --- | --- | --- | --- | --- | --- | --- | --- |
| **DC** | **++++** | **+++** | **++** | **++** | **++** | **+** | **+** | **++** | **++** | **+** | **++++** | **++++** | **++++** |
| **PCNA** | **+++** | **++** | **+** | **+** | **+** | **+** | **-** | **+** | **+** | **-** | **++++** | **+++** | **+++** |
| **Ki67** | **++** | **+** | **+** | **+** | **+** | **+** | **-** | **+** | **+** | **-** | **++++** | **++** | **++** |

AU-auditory cortex

EC-ectorhinal cortex

IC-insular cortex

ILC-infralimbic cortex

MC-motor cortex

OB-olfactory bulb

PIR-piriform cortex

PLC- prelimbic cortex

PR-perirhinal cortex

RMS-rostral migratory stream

SVZ-subventricular zone

SGL-subgranular layer

TA-temporal association cortex

**Semi-quantitative data analysis**

For semi-quantitative evaluation of DC-labeled neurons five grades were used: 0,

no DC-positive neurons present; -, no cells labeled; +, one or only a few cells; ++, several neurons; +++, a substantial number of neurons; ++++, the majority of the expected number of cells is positive (Verwer et al 2007). The cortical borders were evaluated using the cytoarchitecture of cresyl violet stained sections.
