# Supplementary material for: Widespread Doublecortin Expression in the Cerebral Cortex of the Octodon degus
Source: Front Neuroanat. 2021 Apr 29;15:656882. doi: 10.3389/fnana.2021.656882 (PMC8116662; doi:10.3389/fnana.2021.656882)
Supplement: Supplementary file 3 [file Table_3.DOCX]

Supplementary Figure S1

Six photomicrographs demonstrating Ki67 and PCNA labeled nuclei in the: dentate gyrus, piriform and prelimbic cortices.


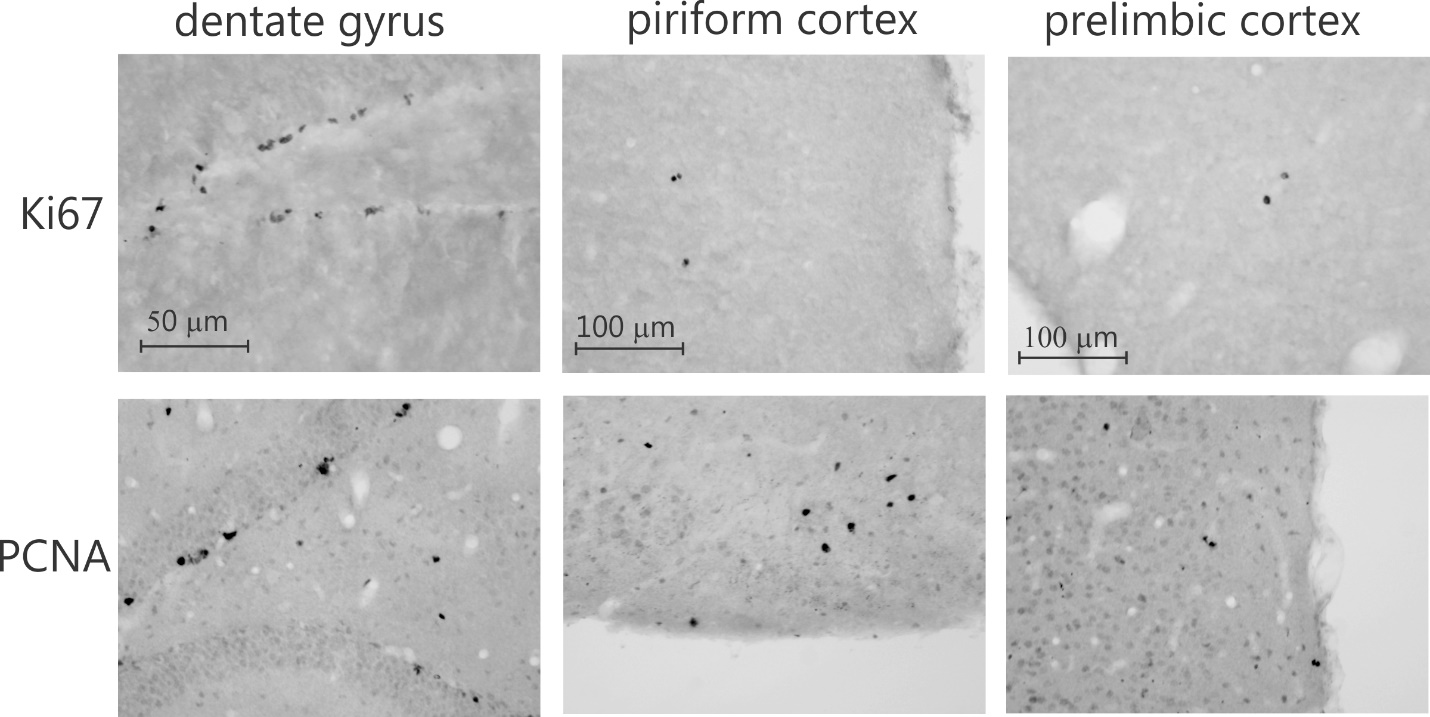


It is of interest to note that many PCNA labeled neurons (and cells) are present in the olfactory bulb (not illustrated). It should be noted that staining of PCNA (and Ki67) is not limited to neuronal cells, it is also present in astrocytes, oligodendrocytes and microglial cells, and there are some labeled cells present in blood vessel walls (not illustrated). Furthermore, a significant number of labeled nuclei is present in layer I. The staining pattern for Ki67 is quite similar to the pattern of labeling described for PCNA, except that the number of labeled cells is even smaller.
