# Supplementary material for: Widespread Doublecortin Expression in the Cerebral Cortex of the Octodon degus
Source: Front Neuroanat. 2021 Apr 29;15:656882. doi: 10.3389/fnana.2021.656882 (PMC8116662; doi:10.3389/fnana.2021.656882)

Supplementary Figure S2

Four photomicrographs demonstrating DCX labeled neuron in the: A, dentate gyrus; B, dorsolateral frontal cortex; C, prelimbic cortex; and D, piriform cortex.


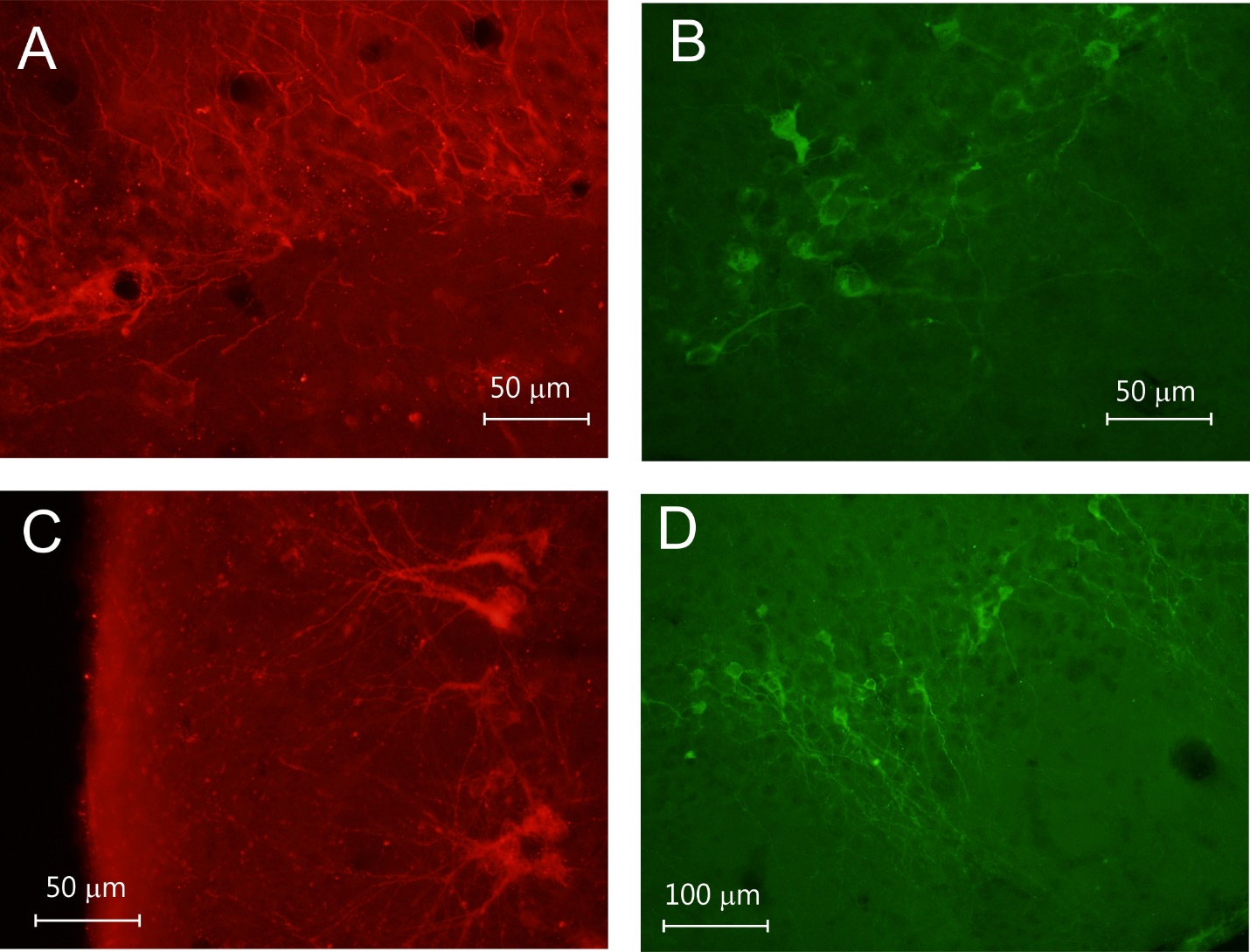

Supplement: Supplementary file 4 [file Table_4.DOCX]
